# High-strength lignin-based carbon fibers *via* a low-energy method†

**DOI:** 10.1039/c7ra10821d

**Published:** 2018-01-02

**Authors:** Zhong Dai, Xiaojuan Shi, Huan Liu, Haiming Li, Ying Han, Jinghui Zhou

**Affiliations:** Liaoning Province Key Laboratory of Plup and Papermaking Engineering, Dalian Polytechnic University Dalian Liaoning Province China zhoujh@dlpu.edu.cn

## Abstract

Bio-renewable carbon fibers are fabricated and employed as high-strength composite materials in many fields. In this work, a facile and low energy consumption method was developed to fabricate high-strength lignin-based carbon fibers. Using iodine treatment, the thermodynamic stability of the lignin-based precursor fibers increased significantly, and thus energy consumption during the preparation of the carbon fibers was reduced. The influence of the iodine treatment on fibers was analyzed by differential scanning calorimetry (DSC), scanning electron microscopy (SEM), Raman spectroscopy, tension testing, *etc.* The resulting iodine lignin-based carbon fibers had better tensile strength (89 MPa) than that of PAN carbon fibers produced by electrospinning technology.

## Abstract 2

A simple low-energy method to fabricate lignin-based carbon fibers with excellent mechanical properties *via* electrostatic spinning.

## Graphical abstract

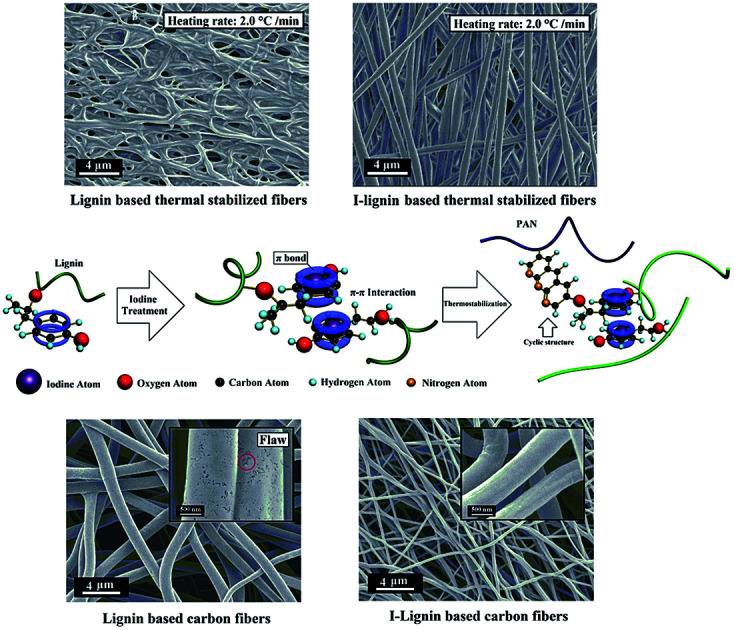

## 1. Introduction

Carbon fibers are an exciting kind of reinforced material, which consist of more than ninety-five percent carbon with a lamellar graphite structure and have been widely used in sporting goods, construction, national defense, and automotive industries. Their excellent physical and chemical properties, such as excellent tensile strength, low density, high creep resistance, and good resistance to chemicals, have attracted tremendous interest.^1–7^ However, the critical issue that limits the practical application of carbon fibers is their high price. Currently, almost 90% of the commercially available carbon fibers are produced using polyacrylonitrile (PAN) as the precursor,^8–12^ which is a non-renewable material and contributes about 50% to the total manufacturing costs of carbon fibers.^13–16^ Therefore, finding an alternative precursor which is bio-renewable and inexpensive is a significant challenge for the carbon fiber industry.^17–20^

Lignin, as the second most abundant natural polymer in the plant cell wall, only next to cellulose, has received increasing attention for use in renewable adhesives,^21^ coatings,^22^ engineering plastics,^23^ and hydrogels.^24^ Unique advantages of lignin including low cost, high carbon content, and an aromatic structure make it an attractive choice as an alternative precursor for the carbon fiber industry.^25–27^ Several strategies have been used to develop the lignin-based carbon fibers (CFs) with novel microstructures and excellent mechanical properties using chemical modification and/or physical blending.^28–32^ To produce the CFs, the section of PAN in lignin-based precursor fibers must undergo thermostabilization to obtain N-containing ladder-type polymers, and carbonization, which removes the other elements aside from carbon and rearranges the carbon bonds to obtain a lamellar graphite structure.^13,14,33^ It is worth noticing that the uniform diameters and lack of structural defects obtained by optimal thermostabilization are critical to increase the mechanical properties of carbon fibers. However, to achieve these good mechanical properties, precursor fibers have a relatively low heating rate and long preparation time during thermostabilization to prevent the melting and deformation of lignin-based stabilized fibers (SFs),^34,35^ which lead to higher energy consumption, compared with just PAN precursor carbon fibers.^24,25^ Therefore, increasing the heating rate and reducing the preparation time in thermostabilization has become a major challenge.

**Scheme 1:**
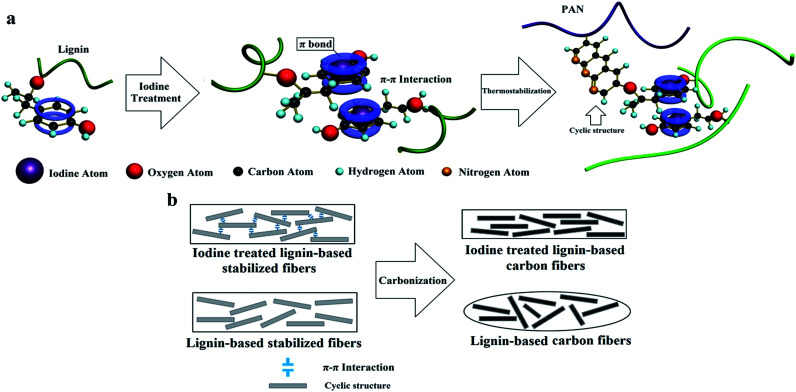
Preparation mechanism of (a) iodine treated lignin and (b) high-strength lignin-based carbon fibers.

Iodine assisted carbonization of biomaterials has been reported in several studies.^28^ However, there are no studies relating to iodine treatment to improve the mechanical properties, the influence of ordered graphitic crystallites, and the defects of interior carbon fibers.In this study, we dissolved lignin and PAN in a suitable solvent, and then the resulting solution was pulled into continuous precursor fibers by electrostatic forces, which was a simple and low-cost method for the preparation of carbon fibers. Subsequently, the precursor fibers were treated by iodine, and finally, the carbon fibers were obtained through thermostabilization and carbonization. The iodine treatment effectively enhanced the rigidity of the lignin chain, which was positive for the increase in morphology retention ability of the precursor fibers during thermostabilization. With a relatively high heating rate during the thermostabilization, the iodine treated lignin-based stabilized fibers (ISFs) have a uniform diameter and no structural defects, compared with the no-iodine SFs. The resulting carbon fibers which were treated with iodine (ICFs) have excellent mechanical properties, even beyond those of the just PAN precursor carbon fibers, which were prepared by the same electrospinning preparation technique. This is a promising method to prepare low-cost, bio-renewable, and high strength carbon fibers for the wider application of carbon fibers. The preparation mechanism for iodine treated lignin and high strength lignin-based carbon fibers is illustrated in Scheme 1.

## 2. Experimental

### 2.1 Materials

Corn residues after enzymatic hydrolysis were purchased from COFCO Corporation. Polyacrylonitrile (PAN, the weight-average molecular weight is 150 000) and iodine crystals were purchased from Sigma-Aldrich. *N*,*N*-Dimethylformamide (DMF) and ethanol were purchased from Sinopharm Chemical Reagent Corporation. All chemicals were used as received.

### 2.2 Preparation of lignin

Corn residues (200 g) and 50% (v/v) ethanol (2000 mL) were mixed in a 3 L three-neck boiling flask, and then the mixture was stirred at 60 °C for 4 h. After cooling, the resulting mixture was filtered, and the filtrate was poured into three volumes of water, the pH was adjusted to 1 and the lignin precipitate was formed. The resulting lignin was washed with deionized water several times, and was then dried in a vacuum.

### 2.3 Electrospinning of precursor fibers

To obtain the electrospinning solutions of lignin–PAN blends, lignin (5 g), PAN (5 g) and DMF (40 g) were mixed in a 100 mL boiling flask. The precursor fibers were manufactured by electrospinning with a working distance of 20 cm, a feed rate of 1.0 mL h^−1^, and an applied voltage power of 20 kV (+15 kV and −5 kV). The resulting precursor fibers were carefully removed from the collector and were dried in a vacuum at 60 °C.

### 2.4 Iodine treatment of precursor fibers

The precursor fibers were placed into a ceramic dish, and then the ceramic dish was put into a jar (4 L) with iodine (10 g). Subsequently, the jar was kept in a 70 °C oven for 12 h to vaporize the iodine. After being cooled, the iodine treated lignin-based precursor fibers (I-precursor fibers) were obtained.

### 2.5 Thermostabilization and carbonization

The precursor fibers were heated to 220 °C under air atmosphere with two heating rates of 0.2 or 2.0 °C min^−1^ in a muffle furnace (KJ-M1200-7LZ, Kejia furnace Co., China) and were kept there for 60 min. Carbonization was performed using a heating rate of 4.0 °C min^−1^ to 1400 °C and held for 120 min in a tube furnace (GSL-1700X, Hefei Kejing materials technology Co., LDT, China).

### 2.6 Measurements

FT-IR spectra were recorded on a Perkin-Elmer Spectrum TWO FT-IR infrared spectrometer in the wavenumber range from 4000 to 400 cm^−1^. X-ray diffraction (XRD) was measured on a Shimadzu XRD-7000S diffractometer with Cu Kα radiation (50 kV, 200 mA, *λ* = 0.154 nm) and a scanning step of 0.02°. The carbon structure of the resulting fibers was analyzed by Raman spectroscopy, and the spectra were recorded on an InVia spectrometer (Renishaw Co., UK) with back-scattered light from a 480 nm laser. Differential scanning calorimetry (DSC) experiments were carried out on a TA Discovery DSC250 at a heating rate of 10 °C min^−1^ under liquid nitrogen. Thermostabilization was assessed on an SDT Q500 analyzer (TA Instruments, USA) with a heating rate of 10 °C min^−1^ under a nitrogen atmosphere. Scanning electron microscopy (SEM) was performed on a JEOL JSM 7800F electron microscope with a primary electron energy of 15 kV and energy dispersive spectrometry (EDS) was performed on an Oxford X-Max 50 spectrometer. The mechanical properties of the carbon fibers were only assessed by uniaxial tensile strength testing using an Instron tension tester (Model 5569, Norwood, USA). Each sample was cut to a length of 4 cm and a width of 1 cm before testing and the strain rate was 2 mm min^−1^. The effective length for the tests was 3 cm.

## 3. Results and discussion

### 3.1 Effects of iodine treatment

**Fig. 1:**
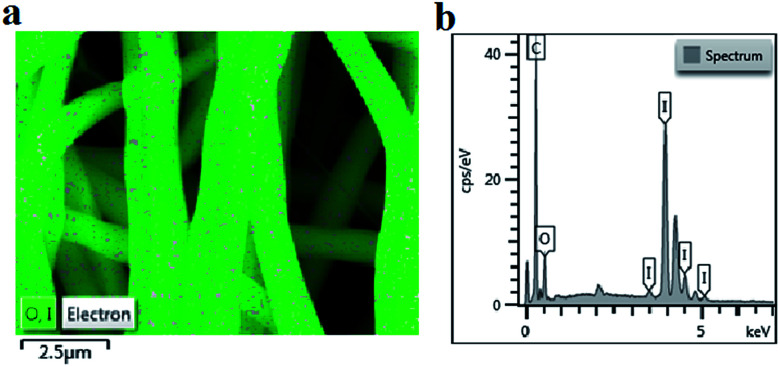
Characterization of the lignin-based precursor fiber: (a) EDS mapping and (b) surface energy spectrum.

Iodine is widely used to form charge transfer complexes with electron-rich molecules, such as aromatic rings. One iodine atom can take a proton from aromatic rings to form a HI molecule at high temperatures, and the other iodine atom can be caught by aromatic rings to form the charge transfer complexes. As shown in Fig. 1a, the iodine element was absorbed into the precursor fibers. To confirm the content of iodine in the precursor fibers, EDS was performed and the results are shown in Fig. 1b. The elemental analysis confirmed the presence of carbon (C, 25.72 wt%), hydrogen (H, 1.88%), nitrogen (N, 5.48%), oxygen (O, 8.54 wt%) and iodine (I, 58.38 wt%) in the I-precursor fibers. These results suggested that iodine treatment was effective in combining the iodine with the aromatic rings of lignin.

**Fig. 2:**
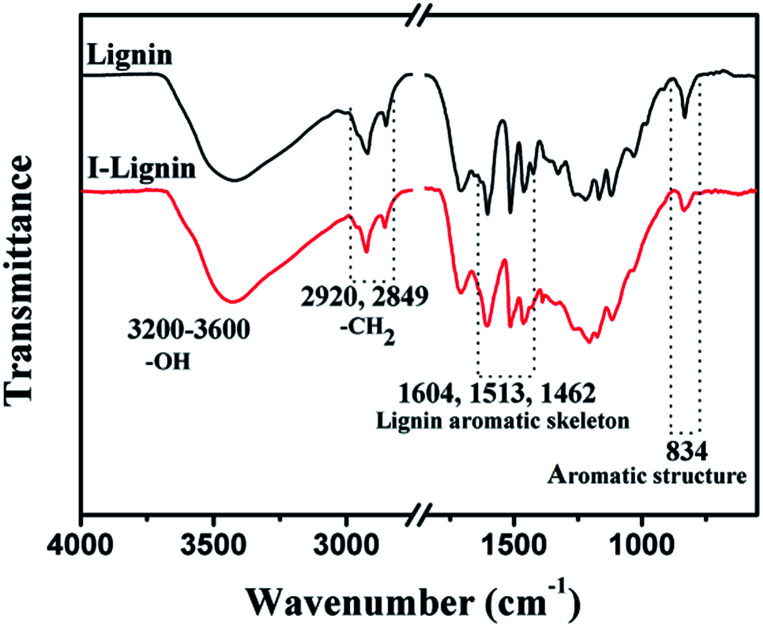
FT-IR spectra of lignin before and after iodine treatment.

The existence of iodine on I-precursor fibers was further confirmed by FT-IR spectra. As shown in Fig. 2a, a typical broad and intense peak appeared from 3200 to 3600 cm^−1^, which was assigned to the O–H stretching in lignin. The peaks at 2920 and 2849 cm^−1^ were assigned to methylene C–H asymmetric stretching and symmetric stretching, respectively. The typical absorbance peaks at 1604, 1513, and 1462 cm^−1^ were assigned to the aromatic skeletal vibrations. The absorption for a benzene ring at 834 cm^−1^ weakened obviously after iodine treatment. This result suggested that the p–π conjugated structure of I-benzene was created during iodine treatment, and was consistent with the EDS findings.^36^

**Fig. 3:**
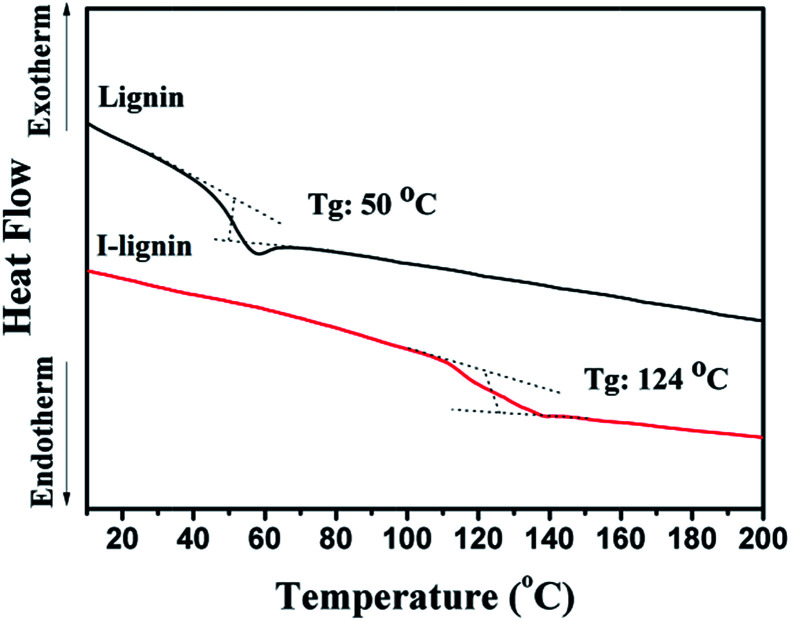
DSC curves of lignin and I-lignin.

Due to the strong electron donating ability of iodine, the charge transfer complexes with iodine had a full π electron orbit, which was effective for enhancing π–π interaction in macromolecules and changed the thermodynamic properties of the macromolecules. As an important thermodynamic property of the macromolecules, the glass transition temperature was reliable and convenient to expound the phase behavior of the materials. As shown in Fig. 3, the glass transition temperature of I-lignin was much higher (124 °C) than that of lignin (50 °C). This result suggested that iodine treatment was effective in enhancing the rigidity of the lignin chain, and thus increasing the thermodynamic stability of lignin.^37^

**Fig. 4:**
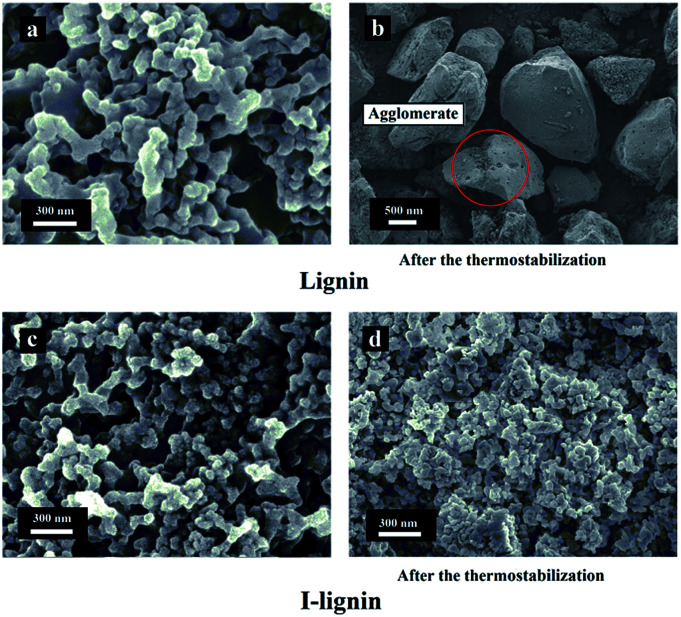
SEM images of (a) lignin, (b) lignin after thermostabilization, (c) I-lignin, and (d) I-lignin after thermostabilization.

The morphologies of lignin and I-lignin (before and after the thermostabilization and heating from room temperature to 220 °C with a heating rate of 1.0 °C min^−1^ under air atmosphere) were observed by SEM. As shown in Fig. 4, the morphology of lignin was similar to that of the I-lignin before iodine treatment. After the thermostabilization, the morphology of lignin changed into a block-like structure. At the same time, I-lignin still kept the original morphology after the thermostabilization, which was mainly attributed to the rigidity of the I-lignin chain, which was positive for preventing the relative motion of the I-lignin chains.

### 3.2 Thermostabilization and carbonization of precursor fibers and I-precursor fibers

**Fig. 5:**
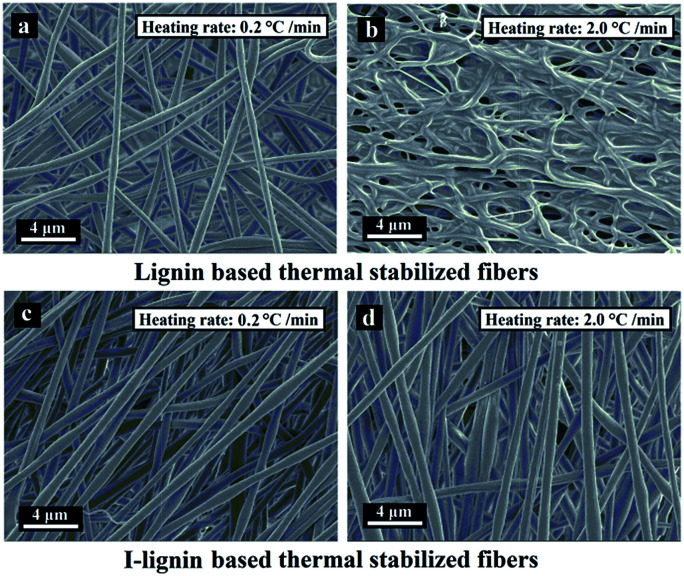
SEM images of the SFs at the heating rates of (a) 0.2 °C min^−1^ and (b) 2.0 °C min^−1^, and ISFs at the heating rates of (c) 0.2 °C min^−1^ and (d) 2.0 °C min^−1^.

The morphologies of the SFs were crucial for enhancing the mechanical strength of the resulting carbon fibers. Structural defects and collapse of the fiber morphology would result in a sharp decline of mechanical strength.^38^ The morphologies of the SFs and ISFs are shown in Fig. 5. The SFs and ISFs showed different fibrous morphology retention *via* the different thermostabilization methods. At the heating rate of 0.2 °C min^−1^, both SFs and ISFs maintained their original morphologies. However, at the heating rate of 2.0 °C min^−1^, the morphologies of the SFs were collapsed, and the ISFs still kept the original morphologies. This result suggested that I-lignin was effective in maintaining the morphology of the I-precursor fibers during thermostabilization with a high heating rate. Furthermore, the morphology of the ISFs was further examined using SEM images at the heating rates of 3.0, 4.0, 5.0, and 6.0 °C min^−1^ in Fig. S1.† The preparation mechanism of the ISFs is illustrated in Scheme 1a.

**Fig. 6:**
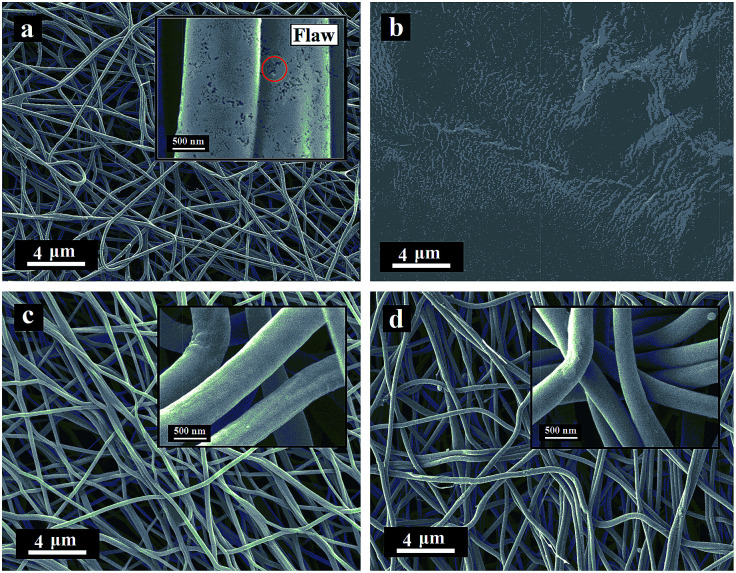
SEM images of the CFs thermally stabilized at the heating rate of (a) 0.2 °C min^−1^ and (b) 2.0 °C min^−1^ and ICFs thermally stabilized at the heating rate of (c) 0.2 °C min^−1^ and (d) 2.0 °C min^−1^.

**Fig. 7:**
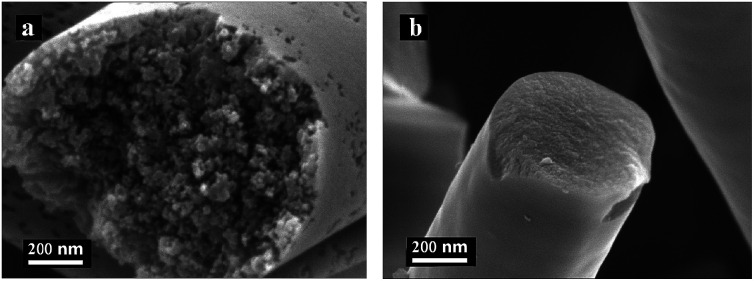
SEM images of the cross-sections of the CFs thermally stabilized at the heating rate of (a) 0.2 °C min^−1^ and ICFs thermally stabilized at the heating rate of (b) 0.2 °C min^−1^.

The SFs and ISFs were carbonized at 1400 °C in a tube furnace under nitrogen atmosphere at a heating rate of 4 °C min^−1^. The morphologies of the CFs and ICFs are shown in Fig. 6. In addition, the cross-sections of the CFs and ICFs are shown in Fig. 7. The CFs undergoing thermal stabilization at the heating rate of 0.2 °C min^−1^ (CFs-0.2) had many structural defects (Fig. 6a and 7a) and the CFs thermally stabilized at the heating rate of 2.0 °C min^−1^ (CFs-2.0) collapsed thoroughly (Fig. 6b). However, the ICFs thermally stabilized at the heating rates of 0.2 and 2.0 °C min^−1^ (ICFs-0.2 and ICFs-2.0) still kept the original morphologies and had no structural defects (Fig. 6c and d and 7b). This result demonstrated that the I-lignin was also effective in maintaining the morphology of ISFs during carbonization. The yield of CFs-0.2, CFs-2.0, ICFs-0.2, and ICFs-2.0 was 37.82, 21.83, 60.84, and 58.59%, respectively. The preparation mechanism of the ICFs and CFs is illustrated in Scheme 1b.

**Fig. 8:**
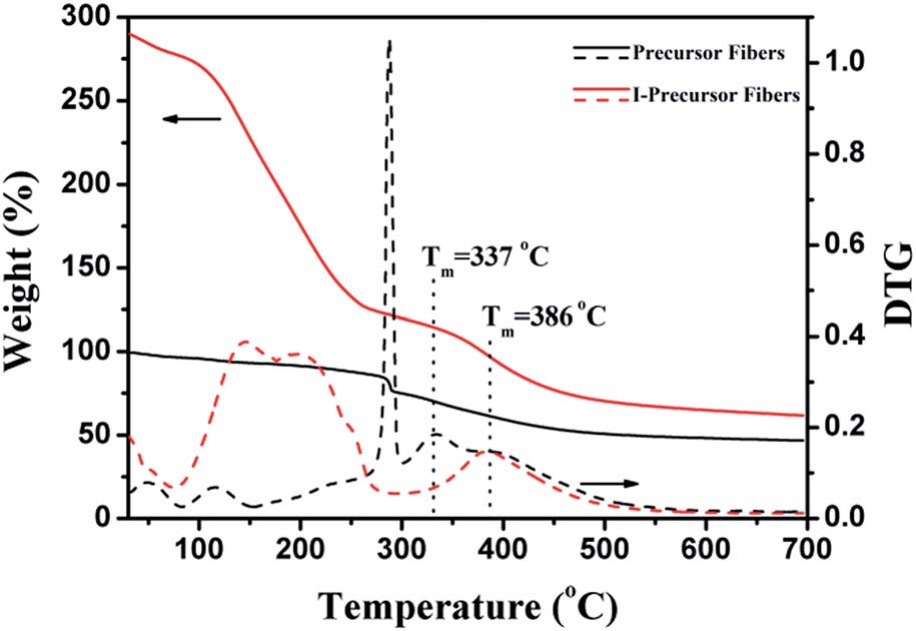
TG-DTG curves of lignin based precursor fibers and I-lignin based precursor fibers.

The TG-DTG curves of precursor fibers and I-precursor fibers are shown in Fig. 8, with the weight of the precursor fibers before iodine treatment being set to 100%. There were two degradations around 50 °C and 117 °C, which were attributed to the evaporation of water and DMF respectively in the TG-DTG curve of the I-precursor fibers. With the increase of temperature, the precursor fibers begin to degrade at 270 °C, which was assigned to the cyclo-dehydrogenation of PAN (Fig. S2†) as well as the degradation of β-O-4. The degradation from 300 to 500 °C was attributed to the decomposition of lignin and reached a maximum value at about 337 °C. The productive rate after complete thermal degradation was 46.4%. For the I-precursor fibers, there was a large weight loss beginning at 80 °C, which was attributed to the removal of iodine compounds, such as I_2_, CH_3_I, and C_2_H_5_I.^28,36,39^ The initial degradation of the C–C chains of lignin in the I-precursor fibers began at 330 °C and the temperature at which there was maximum degradation was about 386 °C, which was higher than that of the precursor fibers. The remaining fraction after complete thermal degradation was 61.4%. As a result, the weight loss of the ISFs decreased, indicating that the iodine-substitution was the key factor for increasing the yield and obtaining unflawed fiber morphology.

### 3.3 Properties of CFs and ICFs

**Fig. 9:**
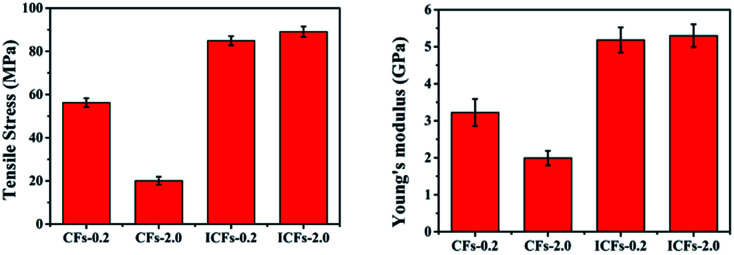
The tensile stress and Young’s moduli of the CFs-0.2, CFs-2.0, ICFs-0.2, and ICFs-2.0.

For the practical application of carbon fibers as an exciting kind of reinforced material, the mechanical properties of CFs-0.2, CFs-2.0, ICFs-0.2, and ICFs-2.0 were described by the tensile stress–strain curves. The sample was cut to a length of 4 cm and width of 1 cm. The effective length for the tests was 3 cm. The stress of the carbon fibers was calculated using the following equation:
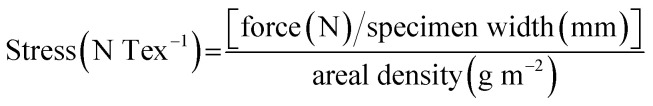


The areal density was the weight of the sample (g) divided by its area (m^2^), and the units of stress (N Tex^−1^) were converted into the tensile stress (GPa) by multiplying the density of the test specimen, which was determined using a Sartorius Secura balance. The tensile stress and Young’s moduli of the CFs-0.2, CFs-2.0, ICFs-0.2, and ICFs-2.0 are shown in Fig. 9. The tensile stress–strain curves of the CFs-0.2, CFs-2.0, ICFs-0.2, and ICFs-2.0 are shown in Fig. S3.† The maximum stress of the fibers significantly increased from 20 to 89 MPa with the iodine treatment. A reasonable explanation is that the unflawed fiber morphology prevented stress concentration, which is the main factor of fiber breakage. However, the Young’s moduli of the ICFs have no significant changes after increasing the heating rate from 0.2 to 2.0 °C min^−1^. It is worth noting that the mechanical strength of the ICFs-2.0 (89 MPa) had exceeded the PAN-based carbon fibers (41 MPa) produced using electrospinning.^40^ The density of the CFs-0.2, CFs-2.0, ICFs-0.2, and ICFs-2.0 was between 1.62 and 1.69 g cm^−3^, which was determined using a Mettler Toledo balance and density kit. The corresponding properties of the carbon fibers are listed in Table 1.

**Table 1:** Properties of the CFs-0.2, CFs-2.0, ICFs-0.2, and ICFs-2.0

Sample	Density (g cm^−3^)	Tensile strength (MPa)	Young’s modulus (GPa)	Strain at break (%)	Yield (%)
CFs-0.2	1.62	56 ± 2	3.2 ± 0.4	1.05 ± 0.05	37.82
CFs-2.0	1.64	20 ± 2	2.0 ± 0.2	0.94 ± 0.02	21.83
ICFs-0.2	1.65	85 ± 2	5.2 ± 0.3	1.00 ± 0.04	60.84
ICFs-2.0	1.69	89 ± 3	5.3 ± 0.3	1.14 ± 0.03	58.5

**Fig. 10:**
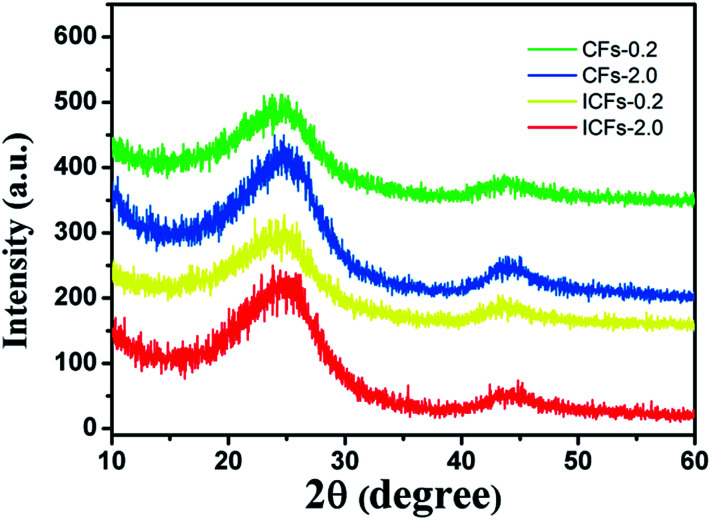
The XRD patterns of the CFs-0.2, CFs-2.0, ICFs-0.2, and ICFs-2.0.

XRD patterns of carbon fibers were produced to characterize the crystalline structures of carbonaceous materials, as presented in Fig. 10. Two broad diffraction peaks at 2*θ* of 24° and 43° were observed, which were attributed to the crystallographic plane of (002) and (100) in the graphite structure, respectively. In addition, the broad peak at 2*θ* of 24° indicated that the sizes of the graphite crystallites possibly were very small and the disordered carbonaceous structure probably was relatively high, probably due to the fact that 4–7% nitrogen, oxygen and other non-carbon impurity atoms still existed in the fibers after carbonization.^41,42^ According to the Bragg method, the average interplanar crystal spacings for (002) in the CF-0.2, CF-2.0, ICF-0.2, and ICF-2.0 samples were calculated to be 0.3711, 0.3630, 0.3573 and 0.3528 nm, respectively. It was found that the average interplanar spacing in the ICFs was lower than that in the CFs, which was due to the increase of the density of fibers.^27^ The difference in the porosity of the fiber mats also caused the density to change. Furthermore, it suggested that the pores were formed in untreated fibers according to Fig. 6 and these disordered structures were harmful for the mechanical properties of carbon fibers.^42,43^

**Fig. 11:**
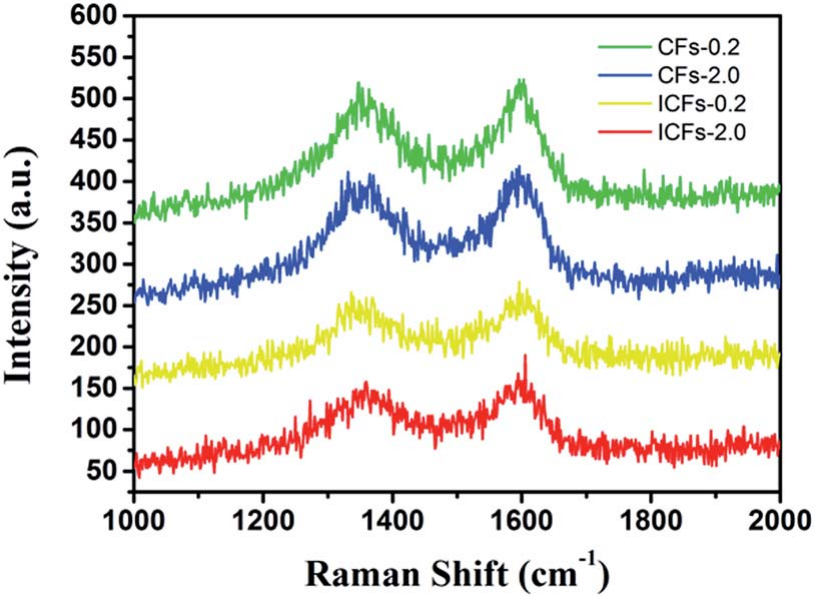
The Raman spectra of the CFs-0.2, CFs-2.0, ICFs-0.2, and ICFs-2.0.

Raman spectroscopy was used to determine the structure of the carbon fibers (Fig. 11). The two major broad peaks centering at about 1345 and 1598 cm^−1^ in the spectrum were referred to as the D and G band, respectively. The D band was related to disordered carbonaceous structure while the G band was related to ordered graphitic structures. The D band was attributed to a hybridized vibrational mode related to graphene layer edges, indicating the number of defects in the graphitic structure, and the G band originated from an in-plane stretching mode of sp^2^ carbon bonds existing in the ideal graphitic lattice. The “*R*-value” of the “D-band” to “G-band” is often used to indicate the degree of structural disorder in carbonaceous materials. The *R*-values of CFs-0.2, CFs-2.0, ICFs-0.2, and ICFs-2.0 were 1.03, 0.94, 0.91, and 0.85, respectively. With the increase in the heating rate during thermostabilization (from 0.2 to 2.0 °C min^−1^), the *R*-value decreased. This suggested that some disordered carbonaceous structures were converted to ordered graphitic crystallites with the increased heating rate during thermostabilization. It is worth noting that the amount of ordered graphitic crystallites also increased after iodine treatment. As a result, iodine treatment on lignin based precursor fibers can improve the mechanical properties of fibers by increasing the content of ordered graphitic crystallites.^44^ This observation was consistent with the SEM results, mechanical properties, and XRD results.

## 4. Conclusion

We have developed a simple low-energy method to fabricate lignin-based carbon fibers with excellent mechanical properties *via* electrostatic spinning. As a result, the study on the effects of iodine treatment on lignin-based carbon fibers suggests that the energy consumption during the preparation of high-strength carbon fibers reduces significantly with the increase of rigidity in the lignin chain. This finding is of great significance to the development of the green carbon fiber industry. These high-strength lignin-based carbon fibers are the more promising candidate instead of the PAN-based carbon fibers used in civilian areas, such as sporting goods and electrodes.

## Conflicts of interest

There are no conflicts to declare.

## Supplementary Material

RA-008-C7RA10821D-s001

## References

[cit1] Poursorkhabi V. , Mohanty A. and Misra M., AIP Conference Proceedings, AIP Publishing, 2015, vol. 1664(1), p. 150003

[cit2] Kai D., Tan M. J., Chee P. L., Chua Y. K., Yap Y. L., Loh X. J. (2016). Green Chem..

[cit3] Dong Kwon Seo J. P. J., Bin Kim H., Kang P. H. (2011). Rev. Adv. Mater. Sci..

[cit4] Ruiz-Rosas R., Bedia J., Lallave M., Loscertales I. G., Barrero A., Rodríguez-Mirasol J., Cordero T. (2010). Carbon.

[cit5] Chatterjee S., Clingenpeel A., McKenna A., Riosa O., Johs A. (2014). RSC Adv..

[cit6] Dong X. Z., Lu C. X., Zhou P. C., Zhang S. C., Wang L. Y., Li D. H. (2015). RSC Adv..

[cit7] Aoki K., Usui Y., Narita N., Ogiwara N., Iashigaki N., Nakamura K., Kato H., Sano K., Ogiwara N., Kametani K., Kim C., Taruta S., Kim Y. A., Endo M., Saito N. (2009). Small.

[cit8] Park S. J. and Lee S. Y., Carbon Fibers, Springer Netherlands, 2015, vol. 210, pp. 1–30

[cit9] Li Q., Xie S. X., Serem W. K., Naik M. T., Li L., Yuan J. S. (2017). Green Chem..

[cit10] Li M., Chang Y., Han G., Yang B. (2011). J. Power Sources.

[cit11] Karpova S. G., Iordanskii A. L., Motyakin M. V., Ol’khov A. A., Staroverova O. V., Lomakin S. M., Shilkina N. G., Rogovina S. Z., Berlin A. A. (2015). Polym. Sci., Ser. A.

[cit12] Dong Y., Lin H., Jin Q., Li L., Wang D., Zhou D., Qu F. (2013). J. Mater. Chem. A.

[cit13] Kubo S., Kadla J. F. (2005). J. Polym. Environ..

[cit14] Sudo K., Shimizu K. (1992). J. Appl. Polym. Sci..

[cit15] Xue B. L., Wen J. L., Xu F., Sun R. C. (2013). J. Appl. Polym. Sci..

[cit16] Darren T. G. R., Baker A. (2013). J. Appl. Polym. Sci..

[cit17] Kadla J. F., Kubo S., Venditti R. A., Gilbert R. D., Compere A. L., Griffith W. (2002). Carbon.

[cit18] Berenguer R., García-Mateos F. J., Ruiz-Rosas R., Cazorla-Amorós D., Morallón E., Rodríguez-Mirasol J., Corderoa T. (2016). Green Chem..

[cit19] Sen S., Patil S., Argyropoulos D. S. (2015). Green Chem..

[cit20] Kai D., Jiang S., Low Z. W., Loh X. J. (2015). J. Mater. Chem. B.

[cit21] Ghaffar S. H., Fan M. (2014). Int. J. Adhes. Adhes..

[cit22] Luo J., Liu Y., Yang S., Flourat A. L., Allais F., Han K. (2017). J. Phys. Chem. Lett..

[cit23] Datta J., Parcheta P., Surówka J. (2017). Ind. Crops Prod..

[cit24] Diao B., Zhang Z., Zhu J., Li J. (2014). RSC Adv..

[cit25] Cybulska I., Brudecki G., Rosentrater K., Julson J. L., Lei H. (2012). Bioresour. Technol..

[cit26] Guo Y. Z., Zhou J. H., Wen J. L., Sun G. W., Sun Y. G. (2015). Ind. Crops Prod..

[cit27] Frank E., Steudle L. M., Ingildeev D., Sporl J. M., Buchmeiser M. R. (2014). Angew. Chem., Int. Ed..

[cit28] Schreiber M., Vivekanandhan S., Mohanty A. K., Misra M. (2015). ACS Sustainable Chem. Eng..

[cit29] Sun Q., Khunsupat R., Akato K., Tao J., Labbé N., Gallego N. C., Bozell J. J., Rials T. G., Tuskan G. A., Tschaplinski T. J., Naskar A. K., Pu Y., Ragauskas A. J. (2016). Green Chem..

[cit30] Chatterjee S., Clingenpeel A., McKenna A., Rios O., Johs A. (2014). RSC Adv..

[cit31] Wang B., Cheng J., Wu Y., Wang D., He D. (2013). J. Mater. Chem. A.

[cit32] Wen J. L., Xue B. L., Xu F., Sun R. C. (2013). J. Appl. Polym. Sci..

[cit33] Poursorkhabi V., Mohanty A. K., Misra M. (2015). J. Appl. Polym. Sci..

[cit34] Mainka H., Täger O., Körner E., Hilfert L., Busse S., Edelmann F. T., Herrmann A. S. (2015). J. Mater. Res. Technol..

[cit35] Wang S., Li Y., Xiang H., Zhou Z., Chang T., Zhu M. (2015). Compos. Sci. Technol..

[cit36] Miyajima N., Dohi S., Akatsu T., Yamamoto T., Yasuda E., Tanabe Y. (2002). Carbon.

[cit37] Li L., Valenzuela-Martinez C., Redondo M., Juneja V. K., Burson D. E., Thippareddi H. (2012). J. Food Sci..

[cit38] Reneker D. H., Yarin A. L. (2008). Polymer.

[cit39] Kajiura H., Tanab̧E Y., Yasuda E. (1997). Carbon.

[cit40] Ding R., Wu H., Thunga M., Bowler N., Kessler M. R. (2016). Carbon.

[cit41] Goudarzi A., Lin L. T., Ko F. K. (2014). J. Nanotechnol. Eng. Med..

[cit42] Lai C., Zhou Z., Zhang L., Wang X., Zhou Q., Zhao Y., Wang Y., Wu X. F., Zhu Z., Fong H. (2014). J. Power Sources.

[cit43] Rials T. G., Baker D. A. (2013). J. Appl. Polym. Sci..

[cit44] Youe W. J., Lee S. M., Lee S. S., Lee S. H., Kim Y. S. (2016). Int. J. Biol. Macromol..

