# Supplementary material for: High-strength lignin-based carbon fibers via a low-energy method
Source: RSC Adv. 2018 Jan 2;8(3):1218–24. doi: 10.1039/c7ra10821d (PMC9076998; doi:10.1039/c7ra10821d)
Supplement: RA-008-C7RA10821D-s001 [file RA-008-C7RA10821D-s001.pdf]

Electronic Supplementary Material (ESI) for RSC Advances.
This journal is © The Royal Society of Chemistry 2017

Supporting Information

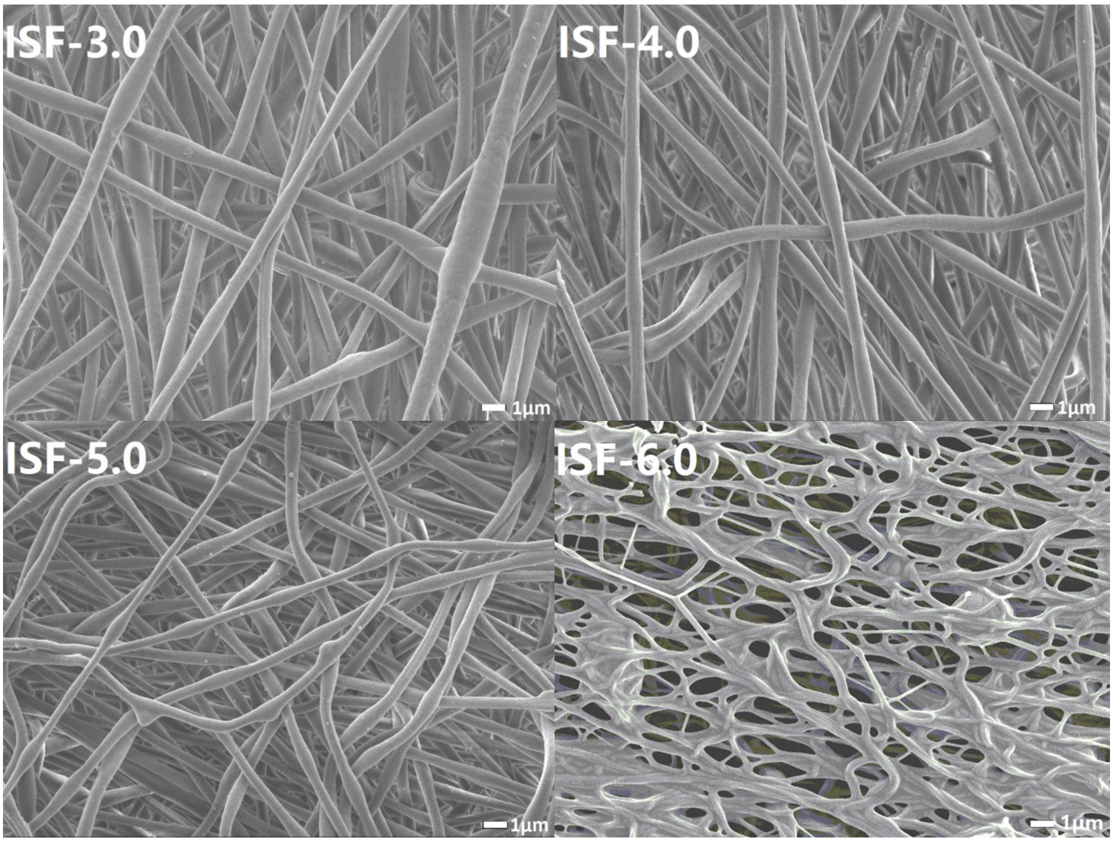

Fig. S1. SEM images of ISFs at the heating rate of 3.0 4.0 5.0 and 6.0 °C/min, respectively.

The fibrous morphologies of ISF was no significantly changed while the heating rate reached to 4.0 ^{o}C/min. However, this morphology has changed when the heating rate elevated to 5.0 ^{o}C/min, it have some teardrop-shaped solid mass come into fiber surface. And the fibrous morphologies were eventually destroyed after the heating rate increased to 6.0 ^{o}C/min.

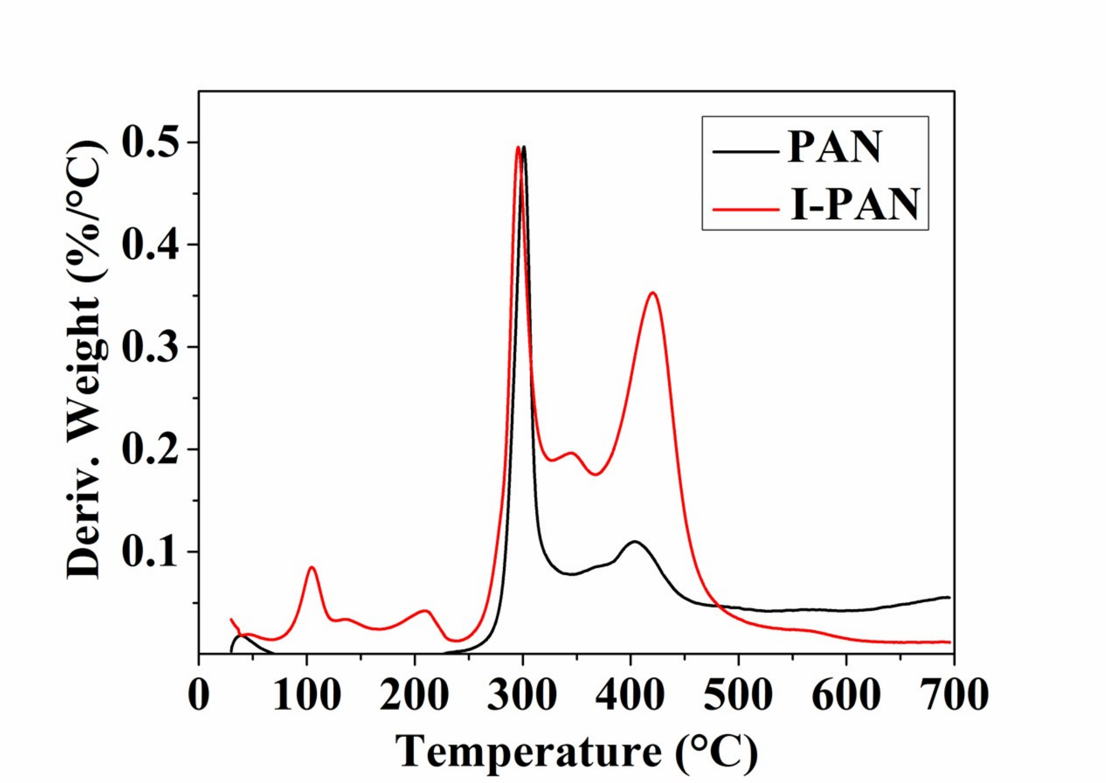

Fig. S2 DTG curves of PAN and I-PAN based precursor fibers.

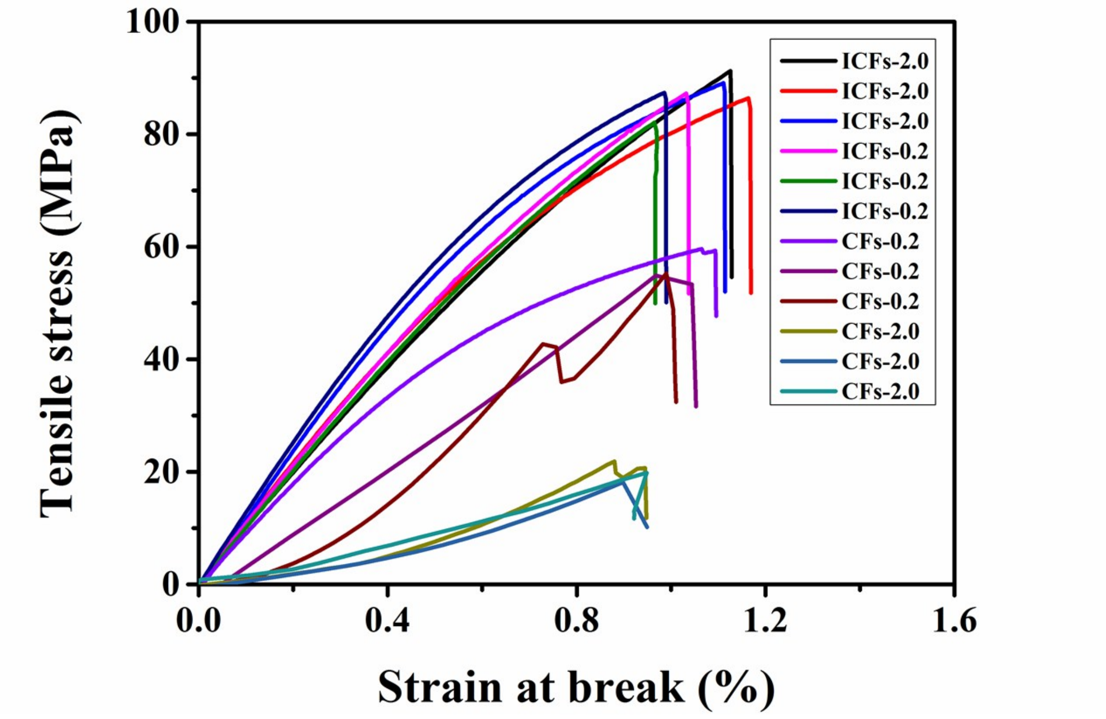

Fig. S3 The tensile stress-strain curves of the CFs-0.2, CFs-2.0, ICFs-0.2, and ICFs-2.0.
